# Supplementary material for: Unveiling IL-33/ST2 Pathway Unbalance in Cardiac Remodeling Due to Obesity in Zucker Fatty Rats
Source: Int J Mol Sci. 2023 Jan 19;24(3):1991. doi: 10.3390/ijms24031991 (PMC9916239; doi:10.3390/ijms24031991)
Supplement: Supplementary file 1 [file ijms-24-01991-s001.zip › ijms-2092282-supplementary.pdf]

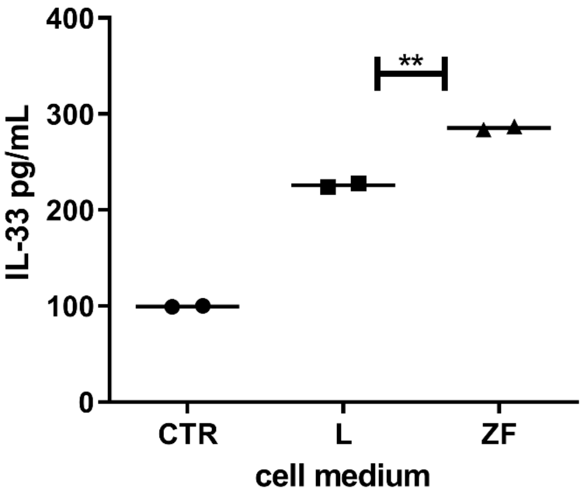

Supplementary Fig S1: characterization of VAT secretome. Conditioned medium from VAT tissue from obese rats contained higher levels of IL-33 protein as measured through ELISA, p= Student's t-test: ** $p < 0.01$.

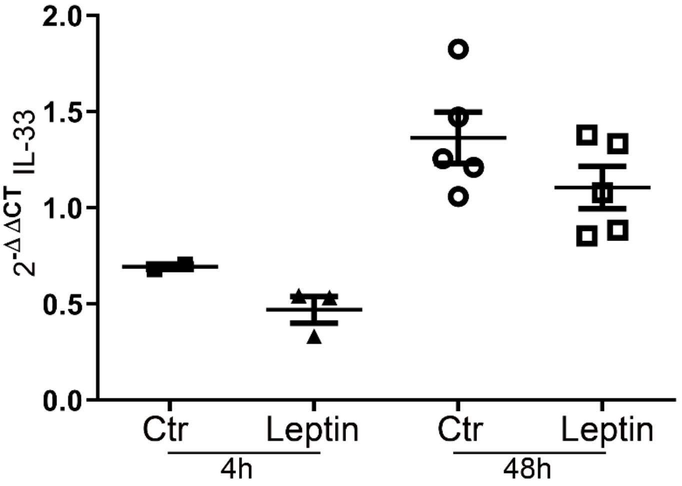

Supplementary Fig S2: Leptin *in vitro* treatment of H9C2 cells modulated IL-33/ST2 signaling. Graphs showed qPCR of H9C2 cell treated with leptin or vector at 4 or 48h. Leptin treatment reduced *IL-33*. Student's t-test: * $p < 0.05$; ** $p < 0.01$, *** $p < 0.001$.
